# Impact of an Educational Program to Reduce Healthcare Resources in Community-Acquired Pneumonia: The EDUCAP Randomized Controlled Trial

**DOI:** 10.1371/journal.pone.0140202

**Published:** 2015-10-13

**Authors:** Jordi Adamuz, Diego Viasus, Antonella Simonetti, Emilio Jiménez-Martínez, Lorena Molero, Maribel González-Samartino, Elena Castillo, María-Eulalia Juvé-Udina, María-Jesús Alcocer, Carme Hernández, María-Pilar Buera, Asunción Roel, Emilia Abad, Adelaida Zabalegui, Pilar Ricart, Anna Gonzalez, Pilar Isla, Jordi Dorca, Carolina Garcia-Vidal, Jordi Carratalà

**Affiliations:** 1 Department of Infectious Diseases, Hospital Universitari de Bellvitge, IDIBELL, Barcelona, Spain; 2 Department of Nursing, Hospital Universitari de Bellvitge, IDIBELL, Barcelona, Spain; 3 School of Health Science, Blanquerna-Ramon Llull University, Barcelona, Spain; 4 Clinical Research and Biotechnology Groups, Faculty of Medicine, Universidad del Norte, Barranquilla, Colombia; 5 School of Nursing, University of Barcelona, IDIBELL, Barcelona, Spain; 6 Institut Català de la Salut, Barcelona, Catalunya, Spain; 7 Department of Nursing, Hospital Clínic de Barcelona, IDIBAPS, Barcelona, Spain; 8 Department of Nursing, Hospital Universitari Arnau de Vilanova, IRBLleida, Lleida, Spain; 9 Department of Respiratory Medicine, Hospital Universitari de Bellvitge, IDIBELL, Barcelona, Spain; 10 Faculty of Medicine, Department of Clinical Sciences, University of Barcelona Barcelona, Spain; University of Ottawa, CANADA

## Abstract

**Background:**

Additional healthcare visits and rehospitalizations after discharge are frequent among patients with community-acquired pneumonia (CAP) and have a major impact on healthcare costs. We aimed to determine whether the implementation of an individualized educational program for hospitalized patients with CAP would decrease subsequent healthcare visits and readmissions within 30 days of hospital discharge.

**Methods:**

A multicenter, randomized trial was conducted from January 1, 2011 to October 31, 2014 at three hospitals in Spain. We randomly allocated immunocompetent adults patients hospitalized for CAP to receive either an individualized educational program or conventional information before discharge. The educational program included recommendations regarding fluid intake, adherence to drug therapy and preventive vaccines, knowledge and management of the disease, progressive adaptive physical activity, and counseling for alcohol and smoking cessation. The primary trial endpoint was a composite of the frequency of additional healthcare visits and rehospitalizations within 30 days of hospital discharge. Intention-to-treat analysis was performed.

**Results:**

We assigned 102 patients to receive the individualized educational program and 105 to receive conventional information. The frequency of the composite primary end point was 23.5% following the individualized program and 42.9% following the conventional information (difference, -19.4%; 95% confidence interval, -6.5% to -31.2%; P = 0.003).

**Conclusions:**

The implementation of an individualized educational program for hospitalized patients with CAP was effective in reducing subsequent healthcare visits and rehospitalizations within 30 days of discharge. Such a strategy may help optimize available healthcare resources and identify post-acute care needs in patients with CAP.

**Trial Registration:**

Controlled-Trials.com ISRCTN39531840

## Introduction

Community-acquired pneumonia (CAP) is a major cause of death and has the highest mortality of all infectious diseases in industrialized countries [1]. In addition, CAP accounts for more than 1 million hospitalizations annually, costing the United States more than $9.7 billion [2]; in Europe, pneumonia costs €10.1 billion annually, with inpatient care costing €5.7 billion, outpatient care costing €0.5 billion, and medication costing €0.2 billion [3]. Currently, 30%-60% of patients diagnosed with CAP are admitted to hospital and the high cost of treating CAP has raised interest in the development of strategies to reduce the length of hospitalization and increase the number of patients who receive care at home [4,5].

Recent studies have found that additional healthcare interactions within 30 days of discharge are common among patients with CAP [6,7], occurring at rates of 7% to 34% [6–9]. The risk factors associated with additional healthcare visits and rehospitalizations typically include comorbid diseases (mainly cardiopulmonary disease), lack of proper information before discharge, and lifestyle factors (mainly unemployment, low level education, and smoking and alcohol abuse) [6,7,9,10].

In an era of increasing competition for economic resources in medical care, institutions have an interest in developing new tools to decrease healthcare resources consumption and to improve healthcare quality [3,11,12]. Discharge planning has been associated with improved use of post-discharge services and fewer readmissions, and appears to prepare patients and caregivers for post-discharge care [13]. Interestingly, it can be hypothesized that some healthcare interactions might be preventable by adequately educating patients before discharge. However, few studies have examined the effect of educational interventions in patients with CAP [14–16]. Moreover, those that have been performed conclude that discharge planning effectively improved patient knowledge, increased cost-effective use of inpatient beds, and improved patient satisfaction with their care [14–17]. Nevertheless, no trials have examined the effects of educational programs to decrease additional healthcare visits and rehospitalizations after discharge in patients with CAP.

We designed a randomized controlled trial (EDUCAP) to test the hypothesis that implementing an individualized educational program for hospitalized patients with CAP would decrease subsequent healthcare interactions after hospital discharge. The primary endpoint of the trial was defined as the frequency of the composite outcome of additional healthcare visits and rehospitalizations within 30 days of hospital discharge.

## Materials and Methods

### Design and Patients

The EDUCAP randomized trial was conducted at three university hospitals in Catalonia, Spain, between January 1, 2011, and October 31, 2014. The participating hospitals were: the Bellvitge Institute for Biomedical Research (IDIBELL)–Hospital Universitari de Bellvitge–a 700-bed university public hospital; the August Pi i Sunyer Biomedical Research Institute (IDIBAPS)–Hospital Clínic de Barcelona–an 800-bed university public hospital; and the Biomedical Research Institute of Lleida (IRBLleida)–Hospital Universitari Arnau de Vilanova–a 400-bed university public hospital. Written informed consent was obtained from patients or relatives provided in accordance with the Declaration of Helsinki. This study was approved by the institutional review board Ethics Committee of Hospital Universitari de Bellvitge (PR183/09), Hospital Clínic de Barcelona (2010/6145) and Hospital Universitari Arnau de Vilanova (1261).

All immunocompetent patients aged 18 years or older and diagnosed with CAP in the participating hospitals were screened for eligibility at the study onset. CAP was defined as the presence of an infiltrate on chest radiograph plus one or more of the following: fever (temperature, ≥ 38.0°C) or hypothermia (< 35.0°C), new cough with or without sputum production, pleuritic chest pain, dyspnea, and altered breath sounds on auscultation. Patients from nursing homes or long-term care facilities were excluded, as were patients with neutropenia (<500/μL), immunoglobulin deficiencies, HIV infection, those who had undergone transplantation or splenectomy, and those who were receiving immunosuppressant and/or corticosteroid therapy (>20 mg/day of prednisone or equivalent). Furthermore, patients with cognitive deficits or those who did not understand Spanish or Catalan were excluded.

### Randomization

We randomly allocated patients to receive either an individualized educational program or conventional information before discharge. Randomization was performed in computer-generated blocks of ten and stratified by participating hospital; the randomization code was kept by the epidemiologist in a sealed envelope. Either in the emergency department or during hospitalization, patients who met the study criteria and provided written informed consent were randomized by a research nurse, who then opened a sealed, sequentially numbered opaque envelope. Due to the nature of the trial, blinding was not possible. However, the clinical staff managing did not know which patients had been allocated to the conventional information group.

### Outcomes

The primary endpoint of the trial was defined as the frequency of the composite outcome of additional healthcare visits and rehospitalizations within 30 days of hospital discharge. This variable included a) visits to a primary care centre because of questions or complications related to CAP (scheduled follow-up visits were excluded), b) emergency department visits for any reason, and c) hospital readmission for any reason. Secondary outcomes included the time to return to activities of daily living, degree of satisfaction with the information received, and the achievement of the educational program’s goals (i.e., patient fluid intake, adherence to drug therapy and preventive vaccines, knowledge and management of the disease, progressive adaptive physical activity, and alcohol and smoking cessation).

### Educational Program, Follow-up and Outcomes Assessment

The individualized educational program was performed according to the Precede-Proceed model for assessing patient health needs and developing discharge planning. This model provides a comprehensive structure for assessing health and quality of life needs, and for designing, implementing, and evaluating health promotion and other public health programs aimed to meet those needs [18]. The individualized educational program was created by the nurses and physicians of the trial coordination team. Two research nurses were trained to deliver the individualized educational program and provide additional educational material. They delivered the individualized educational program to the intervention group in each hospital during patient admission. Patients received the educational program between 24h-72h before hospital discharge, over two sessions of approximately 30 minutes each, and also received a patient education handout about the self-management of CAP. Patients were accompanied by family members and/or caregivers during the educational program. These two sessions were individualized to needs of each patient to have adequate fluid intake, adhere to drug therapy and preventive vaccines, optimize knowledge and management of the disease, use progressive adaptive physical activity, and alcohol and smoking cessation, if required. The interventions in the individualized educational program are fully detailed in an appendix (S2 File).

The control group received conventional information before hospital discharge based on the standard practices of the participating nurses and physicians. The information was not standardized. However, it typically included details about special care needs to be followed at home, information about drug treatment, counseling about smoking and alcohol cessation, and information about healthcare visits. Nurses and physicians also provided this information through the hospital discharge report.

Patients were seen daily by their attending physicians and by at least one of the investigators during their hospital stay. The investigators assessed and recorded all primary and secondary outcome measures. Data collection was conducted at three time points: during hospitalization, at 30 days after discharge, and at 90 days after discharge.

The primary outcome was assessed by searching for hospital readmissions in the electronic health records system (Systems, Applications & Products -SAP-, Waldorf, Germany) of the Catalan Health Service at the three hospitals and confirmed either by asking patients at the 30-day follow-up visit or by telephone. The province of Barcelona (IDIBELL–Hospital Universitari de Bellvitge and the IDIBAPS–Hospital Clínic de Barcelona) and Lleida (IRBLleida–Hospital Universitari Arnau de Vilanova) provides universal health coverage for 5.8 million people [19]. All beneficiaries seen at hospitals in the Catalan Health Service are registered in the SAP, with a unique lifetime personal health number. Data about all hospitalizations and primary care centre or emergency department visits were routinely collected by a research nurse at each centre using a standard protocol, and the process was supervised by the coordination team, which included a qualified infectious diseases physician, who recorded clinical data in a computer-assisted protocol.

The data for secondary outcomes were collected as follows. Time to return to normal activities of daily living was obtained by telephone consultation enquiring about time off work (total number of days lost due to sickness absence) and the Barthel scale 90 days after discharge [20]. Patient satisfaction with the information received was evaluated 30 days after hospital discharge via the question “Are you satisfied with the healthcare information regarding CAP received at discharge?” Responses were recorded on a scale of 1 to 5, from “very unsatisfactory” to “very satisfactory.” Patients were considered satisfied with the information received if the response recorded was 4 or 5. To evaluate the efficacy of the education programs, we assessed patients whether the specific objectives had been achieved prior to discharge, 30 days after discharge (by telephone or at a follow-up visit), and 90 days after discharge (by telephone) using validated scales and questionnaires. Patient fluid intake was evaluated by asking daily water intake. We evaluated drug therapy and preventive vaccine adherence with the Haynes-Sackett test and by checking vaccination histories. Knowledge and management of the disease was evaluated through a CAP knowledge test which was made for the study to assess patient knowledge about the symptoms and complications that can occur. Details of progressive adaptive physical activity were collected by the average daily time walking. Finally, the level of alcohol and smoking cessation or reduction was assessed three months after hospital discharge.

Other clinical and demographic variables were collected by daily monitoring of the patient during hospitalization. Illness severity at presentation was measured using the CURB-65 severity score [21]. Clinical stability and the Charlson comorbidity index were used as described elsewhere [22,23]. All assessments were obtained by a research nurse using a standard protocol with a checklist of items.

### Microbiological Analysis

Samples were obtained according to a standard protocol, and consisted of two sets of blood cultures, a sputum sample when available, urine for antigen detection, and paired acute and convalescent serum samples [24]. *Streptococcus pneumoniae* antigen was detected in the urine using a rapid immunochromatographic assay (BinaxNOW; Binax Inc). *Legionella pneumophila* serogroup 1 antigen was detected in the urine using an enzyme-linked immunosorbent assay (Bartels ELISA, Trinity Biotech). Serological studies were performed by standard methods to determine antibodies against atypical agents.

### Statistical Analysis

Sample size was calculated using the results of a Spanish cohort study of patients admitted with CAP between 2007 and 2009 in IDIBELL (Hospital Universitari de Bellvitge), in which the rate of additional healthcare visits and rehospitalizations was documented as 34.1% [6]. We estimated that a total sample size of 204 patients was needed to detect a 50% reduction in additional healthcare visits and rehospitalizations between the two treatment groups, with an 80% of power and a 5% significance level using the chi-squared bilateral test.

Clinical trial data was summarized using descriptive statistics. Continuous variables were presented as number, mean, standard deviation, range, and median; categorical data, as frequency counts and percentage of subjects per category. We also provided the 95% confidence intervals (CIs) if appropriate.

We compared the primary and secondary outcomes in the two groups. For categorical variables, we performed bivariate analyses using the chi-squared or the Fisher exact tests. For quantitative variables, we performed the Mann–Whitney *U* or the Student *t* test depending on the results of the Kolmogorov–Smirnov test for normality. Percentage differences of the outcomes and mean differences between the two groups, with corresponding 95% confidence intervals, were also computed and presented. Data for the primary and secondary end points were analyzed on an intention-to-treat and per-protocol basis. The intention-to-treat analysis included all randomly assigned patients. Statistical analysis was performed using version 18.0 of the SPSS software package (SPSS Inc., Chicago, Illinois). Statistical significance was established at an *α* value of 0.05. All reported *P* values were based on two-tailed tests.

This study was registered as an International Standard Randomized Controlled Trial, number ISRCTN39531840.

## Results

**Fig 1:**
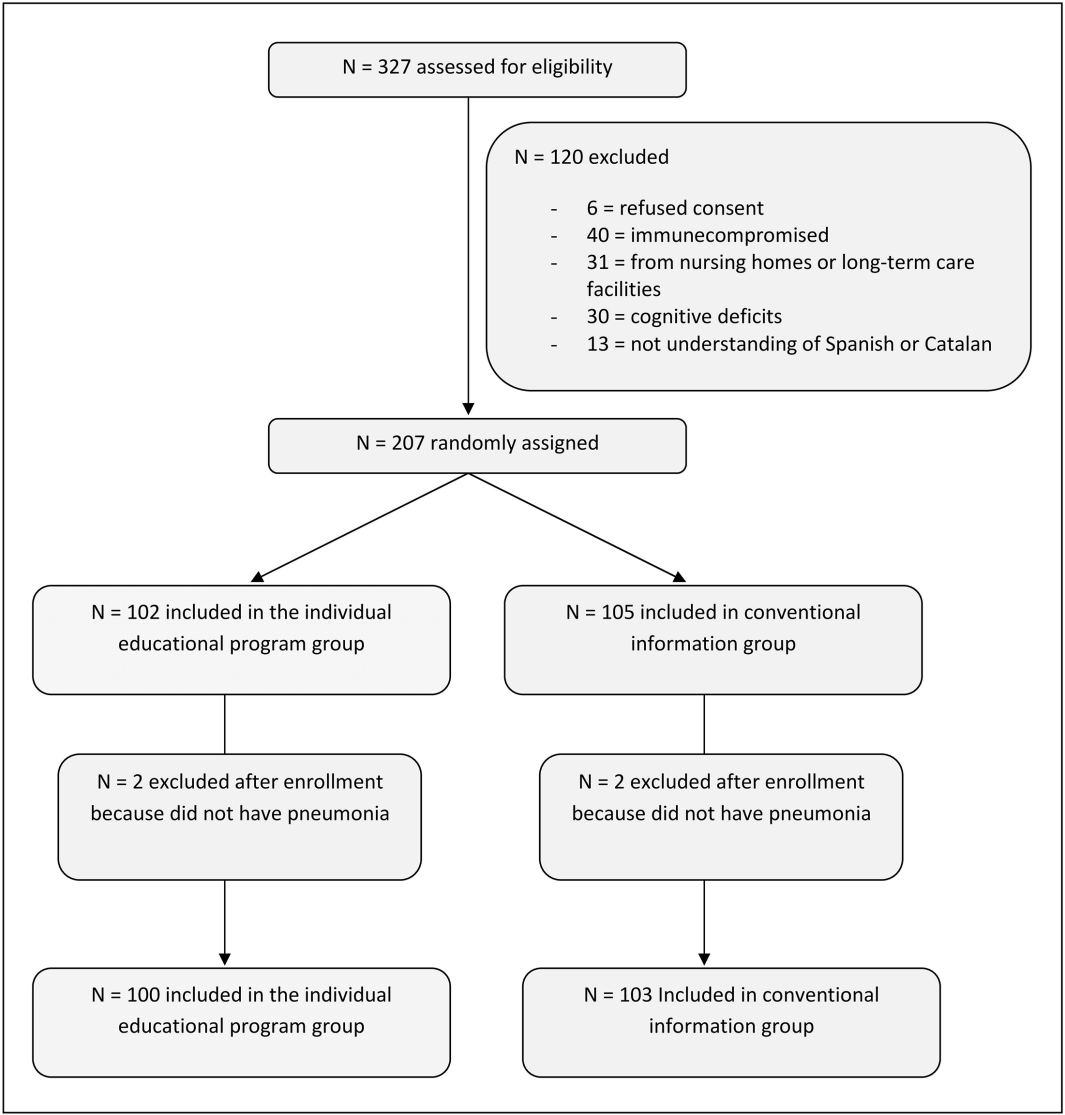
Flowchart of the trial.

We assessed 327 consecutive patients for eligibility, of whom 120 were excluded (Fig 1). A total of 207 patients were randomly assigned and included in an intention-to-treat analysis for the primary and secondary end points. Of these, 102 were assigned to receive the individualized educational program and 105 to receive conventional information. Two participants in each group had alternative diagnoses to CAP after enrollment.

The baseline characteristics of the patients in the two treatment groups were similar (Table 1). No significant differences were found regarding caregiver support at home and the CURB-65 severity score. Complications during hospitalization and rates of intensive care unit admission were similar between the groups. In addition, no significant differences existed between the groups regarding stability at hospital discharge and length of hospitalization. Otherwise, patients in the group receiving conventional information more often were ≥ 70 years old and had none or primary education level (p≤0.05).

**Table 1:** Baseline characteristics. Abbreviations: CAP, community-acquired pneumonia; ICU, intensive care unit; IQR, interquartile range; CURB-65 (confusion, urea > 7 mmol/l, respiratory rate ≥ 30/min, low systolic [<90 mm hg] or diastolic [≤60 mm hg] blood pressure, age ≥ 65 years).

	Individual educational program group		Conventional information group	
	N = 102		N = 105	
Characteristic	No.	(%)	No.	(%)
Male sex	65	(61.9)	59	(57.8)
Age, median (IQR), years^a^	65	(50–77)	72	(59–78)
Age group, years				
21–49	25	(24.5)	17	(16.2)
50–69	36	(35.3)	27	(25.7)
70–97^a^	41	(40.2)	61	(58.1)
Education level				
None or primary-education^a^	58	(56.9)	75	(71.4)
Secondary-education	24	(23.5)	16	(15.2)
Higher-education	12	(11.8)	9	(8.6)
University-education	8	(7.8)	5	(4.8)
Caregiver support home	3	(2.9)	4	(3.8)
Current smoker	31	(30.4)	26	(24.8)
Current drinker	5	(4.9)	7	(6.7)
Influenza vaccination (<1 year)	41	(40.2)	52	(49.5)
Pneumococcal vaccination (<5 years)	14	(13.7)	19	(18.1)
Previous CAP (<1 year)	10	(9.8)	13	(12.4)
Hospitalization within previous 90 days	15	(14.7)	19	(18.1)
Charlson comorbidity index, median (IQR)^a^	2.5	(0–5)	4	(2–6)
CURB-65, median (IQR)	2	(0.8–2)	1	(1–2)
CURB-65 0–1 points	55	(53.9)	47	(44.8)
CURB-65 2 points	34	(33.3)	37	(35.2)
CURB-65 ≥ 3 points	13	(12.7)	21	(20)
Complications during hospitalization^b^	45	(44.1)	34	(32.4)
ICU admission	12	(11.8)	9	(8.6)
Stable on hospital discharge^c^	93	(91.2)	96	(91.4)
Length of hospital stay (days), median (IQR)	6.5	(4–10)	7	(4–10.5)

^a^ P Values≤0.05.

^b^ Related to the pneumonia disease.

^c^ Clinical stability was defined as Halm.

Etiological diagnosis was established in 44 of the 100 patients (44%) receiving individualized education and in 49 of the 103 patients (47.6%) receiving conventional information, excluding patients without pneumonia. The distribution of causative organisms was not different between groups. *Streptococcus pneumoniae* (19 patients receiving individualized education and 31 receiving conventional information) and *Haemophilus influenzae* (7 and 4 patients, respectively) were the most frequently isolated pathogens, followed by *Influenza A* (4 and 3 patients, respectively).

Table 2 details the outcomes for study patients. In the intention-to-treat analysis, the composite outcome of additional healthcare visits and rehospitalization within 30 days of hospital discharge was 23.5% following the individualized educational program and 42.9% following conventional information (difference, -19.4%; 95% CI, -6.5% to -31.2%; p = 0.003) (Table 2). Similar results were obtained in the per-protocol analysis.

**Table 2:** Outcomes for study patients by treatment group. Abbreviations: IQR, interquartile range.

	Individual educational program group		Conventional information group			
	n = 102		n = 105		Difference	p Value^b^
Characteristic	No.	(%)	No.	(%)	(95% CI)^a^	
Primary end point						
Additional healthcare visits and rehospitalization	24	(23.5)	45	(42.9)	-19.4 (-6.5 to -31.2)	0.003
Visits to a primary care centre	13	(12.7)	29	(27.6)	-14.9 (-3.9 to -25.4)	0.009
Emergency department visits	11	(10.8)	27	(25.7)	-14.9 (-4.4 to -25.2)	0.007
Rehospitalization	5	(4.9)	18	(17.1)	-12.2 (-3.7 to -21)	0.007
Secondary end point						
Time to return to activities of daily living						
Time off work (days), median (IQR)	30	(15–66.5)	26	(12.5–37)	4 (59.5 to 68.2)	0.48
Barthel scale, median (IQR)	100	(100–100)	100	(90–100)	0 (97.5 to 93.6)	0.03
Patient satisfied	84	(82.4)	19	(18.4)	64 (51.5 to 73.6)	<0.001
Objectives of educational program						
Proper fluid intake^c^	97	(95.1)	53	(50.5)	44.6 (33.5 to 54.4)	<0.001
Adherence to drug therapy^d^	98	(96.1)	101	(92.2)	-3.9 (-6.3 to 5.9)	1
Influenza vaccination^e^	9	(8.8)	6	(5.8)	3 (-4.3 to 10.8)	0.44
Pneumococcal vaccination^e^	11	(10.8)	8	(7.8)	3 (-4.9 to 11.5)	0.48
Knowledge and management of the disease^f^	100	(98)	21	(20.2)	77.8 (68.1 to 84.7)	<0.001
Progressive adaptive physical activity^g^	82	(80.4)	60	(57.1)	23.3 (10.1 to 34.8)	<0.001
Smoke cessation	15	(50)	6	(23.1)	26.9 (1.5 to 47.6)	0.05
Smoke reduction	23	(76.7)	8	(30.8)	45.9 (19.8 to 64.2)	0.001
Alcohol cessation	2	(40)	1	(14.3)	25.7 (-20.8 to 64.5)	0.52
Alcohol reduction	2	(40)	1	(14.3)	25.7 (-20.8 to 64.5)	0.52

^a^ Values are percentage points for categorical variables.

^b^ Categorical variables were compared using the Fisher exact test and continuous variable using the Mann-Whitney *U* test.

^c^ Proper fluid intake includes patients who drink at least 1,5 liters daily.

^d^ Patients were stratified into good adherence to drug therapy according to the Haynes-Sackett test (80–110% of treatment adherence).

^e^ Influenza and pneumococcal vaccination were evaluated three months after discharge.

^f^ Knowledge and management of the disease was evaluated through CAP knowledge test (sufficient or excellent knowledge).

^g^ Progressive adaptive physical activity was collected by the time walking away schedule (15–30 daily minutes).

Compared with the group that received the individualized educational program, the group that received conventional information had more frequent primary care centre visits (12.7% vs 27.6% [difference, -14.9%; 95% CI, -3.9% to -25.4%; p = 0.009]), emergency department visits (10.8% vs 25.7% [difference, -14.9%; 95% CI, -4.4% to -25.2%; p = 0.007]) and rehospitalizations (4.9% vs 17.1% [difference, -12.2%; 95% CI, -3.7% to -21%; p = 0.007]), within 30 days of hospital discharge. One patient who received conventional information died within 30 days after hospital discharge.

Regarding the secondary end points, the time to return to activities of daily living was similar in the two groups. The median (interquartile range) of the Barthel scale 90 days after hospital discharge was higher in the group receiving individualized education than in the group receiving conventional information, as was patient satisfaction with the information regarding CAP received. In addition, we found a difference in the achievement of the educational program objectives between the groups. Patient fluid intake, knowledge and management of the disease, and progressive adaptive physical activity were higher among those receiving individualized education than among those receiving conventional information. No significant differences were found in adherence to drug therapy, influenza and pneumococcal vaccination uptake, and alcohol cessation rates. However, smoke cessation 90 days after hospital discharge was more frequent in the group that received individualized education (50% vs 23.1% [difference, 26.9%; 95% CI, 1.5% to 47.6%; p = 0.05]).

A post hoc analysis of patients aged 70-years or older was performed. The composite outcome of additional healthcare visits and rehospitalization within 30 days of hospital discharge was 26.8% following the individualized educational program and 47.5% following conventional information (difference, -20.7%; 95% CI, -26.8% to -47.5%; p = 0.04). Compared with the group that received the individualized educational program, the group that received conventional information had more frequent emergency department visits within 30 days of hospital discharge (4% vs 16% [difference, -16.5%; 95% CI, -9.8% to -26.2%; p = 0.045]). However, no differences were found regarding primary care centre visits and rehospitalizations within 30 days of hospital discharge in this group.

## Discussion

In the EDUCAP randomized trial, we assessed an individualized educational program for hospitalized patients with CAP that focused on improving patient fluid intake, adherence to drug therapy and preventive vaccines, knowledge and management of the disease, progressive adaptive physical activity, and counseling for alcohol and smoking cessation. Importantly, we found that this educational program decreased the frequency of healthcare visits and rehospitalizations within 30 days of discharge.

Some studies have concluded that strategies to reduce the length of hospitalization, as well as the trend toward community-based treatment, of patients with CAP should be accompanied by an increased emphasis on the information and support required by patients when returning home [14,15,17]. In addition, it has been documented that hospitalization for CAP is associated with more healthcare interactions after hospital discharge and higher long-term mortality compared with other major medical conditions [6,7,25–28]. Therefore, some investigators have recommended that new educational interventions are needed to improved patient understanding of their post-discharge care. These interventions could have important economic benefits by encouraging cost-effective health service use. Discharge planning has been associated with improved referral to and utilization of post-discharge services, and also with fewer readmissions. Furthermore, such planning appears to prepare patients and caregivers for post-discharge care [13].

Few studies have evaluated the efficacy of educational interventions in patients with CAP. In the studies undertaken to date, the results are mixed. Some studies support the usefulness of such programs in improving patient understanding of post-discharge care [14,15], but others do not [16]. For example, investigators designed interventions to improve patient knowledge that aimed to reduce the time from clinical stability to the switch to oral antibiotics. Patient education included explaining that it takes time to recover from pneumonia, recommending that medications be taken as prescribed, that patient eat healthy foods, and that they monitor for warning signs. Patients were satisfied that they received the information which needed to recover, and most reported that they were of the danger signs of relapse [14]. In the present study, the individualized educational program results in a significant decrease in additional healthcare visits and rehospitalizations within 30-days of hospital discharge.

The group that received the individualized educational program achieved more of the educational objectives. Patient fluid intake, knowledge and management of the disease, progressive adaptive physical activity, and smoking cessation were higher, although no differences were found in adherence to drug therapy, influenza and pneumococcal vaccination uptake, and alcohol cessation. Our findings are consistent with the findings of previous reports that educational interventions improved patient experiences, increased their understanding of post-discharge care, and increases the level of patient satisfaction [14,15]. Moreover, our results may help to developed a new model of in-hospital smoking cessation intervention as suggested in a recent review [29].

The strengths of this study are that it is the first randomized, controlled clinical trial with an intervention arm that received individualized patient education according to the Precede–Proceed model [18]. In addition, a large number of patients were included and just one patient who died was lost of 30-day follow-up visit. Nevertheless, there were no missing data on primary and secondary outcomes. However, some limitations should be acknowledged. First, patients receiving conventional information tended to be older and with low educational level; however, when we restricted the analysis to patients aged 70-years or older and none or primary-education we obtained similar results. In patients aged 70-years or older, the individualized educational program results only have a significant decrease in emergency department visits within 30 days of hospital discharge. Our finding concurs with previous reports that patient education could optimize the use of post-discharge services in patients with CAP [6,15]. Second, information about additional healthcare visits and rehospitalizations within 30 days of discharge were obtained by reviewing the Catalan Health Services database and checked by asking patients or family members at the final outpatient visit or by telephone. Therefore, patients or relatives might not have remembered some visits to private primary care centre or hospitals. Third, we did not evaluate the long-term mortality after hospital discharge in our study. Future studies, may need to assess the effect of individualized educational program on long-term mortality. Finally, it should be emphasized that the EDUCAP trial was not blinded.

In summary, the implementation of an individualized educational program for hospitalized patients with CAP was effective in reducing subsequent healthcare visits and rehospitalizations within 30 days of discharge. Such a strategy may help optimize available healthcare resources and identify post-acute care needs in patients with CAP.

## Supporting Information

S1 File CONSORT Checklist. (DOC)

S2 File Educational program objectives. (DOCX)

S3 File EDUCAP study protocol. (DOCX)
