# Supplementary material for: Impact of an Educational Program to Reduce Healthcare Resources in Community-Acquired Pneumonia: The EDUCAP Randomized Controlled Trial
Source: PLoS One. 2015 Oct 13;10(10):e0140202. doi: 10.1371/journal.pone.0140202 (PMC4603897; doi:10.1371/journal.pone.0140202)
Supplement: S2 File — (DOCX) [file pone.0140202.s002.docx]

**S2.** Educational program objectives

1. **Improve proper fluid intake:** 75% of patients drink at least 2 litters daily hydration during the month after hospital discharge.

| **Specific objective** | **Contents** | **Activities** | **Equipment** | **Indicators** | **Assessment** |
| --- | --- | --- | --- | --- | --- |
| *Knowledge:*  Identify why proper fluid intake is necessary. | General knowledge of the need for proper fluid intake. | Assessment of prior learning and verbal information reinforced with written information (patient education handout). | Patient education handout: Information for patients with pneumonia before hospital discharge. | Knowledge about physiological water requirement.  Norm: 80% of participants.  Assessment tool:  Asking patients at the 30-day visit or by telephone | - Excellent^a^ - Sufficient^b^ - Insufficient^c^ |
| *Skills:*  Drink at least 2 liters of water a day. | Explain it is recommended to drink a few glasses of water each day and the techniques to hydrate properly (example: carry an individual bottle of water). | Assessment of prior learning and verbal information reinforced with written information (patient education handout). | Patient education handout: Information for patients with pneumonia before hospital discharge. | Ability to properly hydrate by drinking at least 2 litters daily.  Norm: 75% of participants.  Assessment tool:  Asking patients or evaluation of the oral mucosa at the 30-day visit or by telephone. | - Excellent > 1,5 liters daily - Sufficient = 1-1,5 liters daily - Insufficient < 1 liter daily |
| *Attitudes:*  Assess the risks and benefits of hydration. | List the risks and benefits of proper hydration. | Assessment of prior learning and verbal information reinforced with written information (patient education handout). | Patient education handout: Information for patients with pneumonia before hospital discharge. | Know the risks and benefits of proper hydration.  Norm: el 80% of participant.  Assessment tool:  *Likert Scale* at the 30-day visit or by telephone. Responses are recorded on a scale of 1 to 5, from “strongly disagree” to “strongly agree”. | - Excellent = 4-5 - Sufficient = 3 - Insufficient <3 |

^a^ Reminds all information.

^b^ Reminds general information.

^c^ Not remember the information.

| **Specific objective 1** | **Contents** | **Activities** | **Equipment** | **Indicators** | **Assessment** |
| --- | --- | --- | --- | --- | --- |
| *Knowledge:*  Identify the indication of drug therapy | Identify the indication of each drug to be followed. | Assessment of prior learning and verbal information reinforced with written information (patient education handout) | Patient education handout: Information for patients with pneumonia before hospital discharge. | Knowledge about drug therapy indications (most importantly, antimicrobial use).  Norm: 80% of participants.  Assessment tool:  Asking patients at the 30-day visit or by telephone. | - Excellent^a^ - Sufficient^b^ - Insufficient^c^ |
| *Skills:*  Taking prescribed medication properly | Techniques to remember to take medication. Information about prescribed dosage and proper drug administration. | Assessment of prior learning and verbal information reinforced with written information (hospital discharge report and patient education handout) | Hospital discharge report and patient education handout: Information for patients with pneumonia before hospital discharge. | Ability to take the prescribed drug properly.  Norm: 80% of participants.  Assessment tool:  *Haynes-Sacket test* at the 30-day visit or by telephone. | - Excellent 90 – 110% - Sufficient 80 – 90% - Insufficient <80% o >110% |
| *Attitudes:*  Assess the risks of not taking medication properly. | List the risks and side effects of not taking drug therapy properly. | Nurse-patient communication. | Oral communication and patient handout. | Know the risks and side effects of not taking drug therapy properly.  Norm: 70-80% of participants.  Assessment tool:  *Likert Scale* at the 30-day visit or by telephone. Responses are recorded on a scale of 1 to 5, from “strongly disagree” to “strongly agree”. | - Excellent = 4-5 - Sufficient = 3 - Insufficient <3 |

1. **Adherence to drug therapy and preventive vaccines:** 80% of patient compliance with adherence to therapy, during the three months after discharge.

^a^ Reminds all information.

^b^ Reminds general information.

^c^ Not remember the information.

| **Specific objective 2** | **Contents** | **Activities** | **Equipment** | **Indicators** | **Assessment** |
| --- | --- | --- | --- | --- | --- |
| *Knowledge:*  Identify the use of  Influenza and pneumococcal vaccination. | Explain the importance of  Influenza and pneumococcal vaccination. | Assessment of prior learning and verbal information reinforced with written information (patient education handout) | Patient education handout: Information for patients with pneumonia before hospital discharge. | Knowledge about the importance of influenza and pneumococcal vaccination.  Norm: el 70 – 80% of participants.  Assessment tool:  Interview at 30 days visit after discharge. | - Excellent^a^ - Sufficient^b^ - Insufficient^c^ |
| *Skills:*  Influenza and pneumococcal vaccination uptake. | Know the timing and where to go for administration of influenza and pneumococcal vaccination. | Assessment of prior learning and verbal information reinforced with written information (patient education handout) | Patient education handout: Information on the discharge of the patient with pneumonia. | Influenza and pneumococcal vaccination uptake.  Norm: el 80% of indicated patient.  Assessment tool:  Checking vaccination histories at the 30 and 90 days after discharge. | Influenza vaccination:   - Yes - No   Pneumococcal vaccination :   - Yes - No |
| *Attitudes:*  Assess influenza and pneumococcal vaccination need. | List the benefits of assess influenza and pneumococcal vaccination. | Nurse-patient communication. | Oral communication and patient handout. | Know the benefits of assess influenza and pneumococcal vaccination.  Norm: el 70 -80% of participants.  Assessment tool:  *Likert Scale* at the 30-day visit or by telephone. Responses were recorded on a scale of 1 to 5, from “strongly disagree” to “strongly agree”. | - Excellent = 4-5 - Sufficient = 3 - Insufficient <3 |

^a^ Reminds all information.

^b^ Reminds general information.

^c^ Not remember the information.

1. **Knowledge and management of the disease:** 70% of patients can self-manage and control warning signs about CAP during the month after hospital discharge.

| **Specific objective 1** | **Contents** | **Activities** | **Equipment** | **Indicators** | **Assessment** |
| --- | --- | --- | --- | --- | --- |
| *Knowledge:*  Know the warning signs and possible complications that may occur in your home and what to do if any of these events happen. | Explain the signs that can occur. | Assessment of prior learning and verbal information reinforced with written information (patient education handout) | Patient education handout: Information on the discharge of the patient with pneumonia | Knowledge about the symptoms and complications that can occur.  Norm: 70 – 80% of participants.  Assessment tool:  Interview at 30 days visit after discharge. | - Excellent^a^ - Sufficient^b^ - Insufficient^c^ |
| *Skills:*  Detection of warning signs and symptoms characteristic of the recovery process, which can be experienced or undergone, and self-management. | Know how to detect and manage the typical signs or complications of the recovery process. For example: fever, cough, etc... | Assessment of prior learning and verbal information reinforced with written information (patient education handout) | Patient education handout: Information on the discharge of the patient with pneumonia.  Practical exercise. | Skills to detect signs and symptoms during recovery process and self-management.  Norm: 70-80% of participants.  Assessment tool:  Practical exercise: relate the symptoms (typical or not typical) and actions. | - Excellent^a^ - Sufficient^b^ - Insufficient^c^ |
| *Attitudes:*  Be able to consult the healthcare system when it is necessary. | Consult the healthcare system in case of appearance of signs that are not typical of the process of convalescence. | Nurse-patient communication. | Oral communication, supported with the patient education handout. | Skill to consult healthcare system in case of appearance non-typical symptoms  Norm: 70% of participants.  Assessment tool:  Assess the reasons for additional healthcare visits at 30 days after discharge. | - Excellent^a^ - Sufficient^b^ - Insufficient^c^ |

^a^ Reminds all information.

^b^ Reminds general information.

^c^ Not remember the information.

| **Specific objective 2** | **Contents** | **Activities** | **Equipment** | **Indicators** | **Assessment** |
| --- | --- | --- | --- | --- | --- |
| *Knowledge:*  Achieve general knowledge about pneumonia. | Basic knowledge about what is pneumonia and how it is produced. | Verbal information reinforced with written information (patient education handout) | Patient education handout: Information on the discharge of the patient with pneumonia | Knowledge about what is pneumonia and how it is produced.  Norm: 70-80% of participants.  Assessment tool:  Interview at 30 days visit after discharge (community-acquired pneumonia knowledge test). | - Excellent^a^ - Sufficient^b^ - Insufficient^c^ |

^a^ Reminds all information.

^b^ Reminds general information.

^c^ Not remember the information.

1. **Progressive adaptive physical activity:** 70% of patients participate a progressive physical activity tailored to their needs during the three months after discharge.

| **Specific objective** | **Contents** | **Activities** | **Equipment** | **Indicators** | **Assessment** |
| --- | --- | --- | --- | --- | --- |
| *Knowledge:*  Know the importance of participating in a progressive physical activity done at patient’s own pace. | Explain the importance of starting the physical activity first while sitting and then begin gradually walking. | Assessment of prior learning and verbal information reinforced with written information (patient education handout). | Patient education handout: Information on the discharge of the patient with pneumonia. | Knowledge about progressive physical activity during recovery process.  Norm: 70-80% of participants.  Assessment tool:  Interview at 30 days visit after discharge. | - Excellent^a^ - Sufficient^b^ - Insufficient^c^ |
| *Skills:*  Know how to do a progressive physical activity during convalescence. | Begin a progressive physical activity at the patient’s own pace during convalescence. | Verbal information. | Oral communication. | Skills to do a progressive physical activity.  Norm: 70% of participants.  Assessment tools:   - - Barthel index*:* baseline, pre-discharge, 30 days after discharge*.*   - Daily physical activity.   Interview 30-days (by telephone or at a follow-up visit) after discharge. | Barthel index:   - Excellent: 61-100 - Sufficient: 41-60 - Insufficient:≤ 40   Walking time:   - Excellent ≥ 30 daily minutes. - Sufficient 15-30 daily minutes. - Insufficient < 15 daily minutes. |
| *Attitudes:*  Assess the need to start a progressive regular physical activity. | List the benefits of progressive physical activity. | Nurse-patient communication. | Oral communication, supported with the patient education handout. | Assess the need to start a progressive physical activity.  Norm: 80% of participants.  Assessment tool:  *Likert Scale* at the 30-day visit or by telephone. Responses are recorded on a scale of 1 to 5, from “strongly disagree” to “strongly agree”. | - Excellent = 4-5 - Sufficient = 3 - Insufficient <3 |

^a^ Reminds all information.

^b^ Reminds general information.

^c^ Not remember the information.

1. **Alcohol and smoking cessation:** 60% of patients begin strategies of alcohol and smoking cessation during three months after hospital discharge.

| **Specific objective 1** | **Contents** | **Activities** | **Equipment** | **Indicators** | **Assessment** |
| --- | --- | --- | --- | --- | --- |
| *Knowledge:*  Know the risks of smoking and assess the need to quit smoking to improve their health. | Explain the importance of smoking cessation. | Assessment of prior learning and verbal information reinforced with written information (patient education handout). | Patient education handout: Information on the discharge of the patient with pneumonia. | Knowledge of the risks of smoking and the need to quit.  Norm: 85% of participants.  Assessment tool:  Questionnaire. | - Excellent^a^ - Sufficient^b^ - Insufficient^c^ |
| *Skills:*  Be able to initiate strategies to quit smoking. | Explain to the patient that there are different techniques to help quit smoking. | Verbal information. | Oral communication. Visual graphics documents. | Skills on different techniques to quit.  Norm: 70-80% of participants.  Assessment tool:  Explain the different techniques. | - Excellent^a^ - Sufficient^b^ - Insufficient^c^ |
| *Attitudes:*  Start looking for help to quit smoking. | Inform the patient where he/she can go to receive help and encouragement. | Counseling, nurse-patient communication and written support. | Counseling, supported with the patient education handout. | Assess whether the patient has initiated strategies to achieve the objective described.  Norm: 60% of participants.  Assessment tool:  Interview 30-days (by telephone or at a follow-up visit) and 3 months (by telephone) after discharge. | Starts strategies?:   - - Yes   - No   Still smoking?:   - - Yes   - Consumption reduce   - No |

^a^ Reminds all information.

^b^ Reminds general information.

^c^ Not remember the information.

| **Specific objective 2** | **Contents** | **Activities** | **Equipment** | **Indicators** | **Assessment** |
| --- | --- | --- | --- | --- | --- |
| *Knowledge:*  Know the risks of alcohol abuse and assess the need to reduce consumption to improve their health. | Explain the importance of reducing alcohol abuse. | Assessment of prior learning and verbal information reinforced with written information (patient education handout). | Patient education handout: Information on the discharge of the patient with pneumonia. | Knowledge about the risks and the need to reduce alcohol consumption.  Norm: 85% of participants.  Assessment tool:  Questionnaire. | - Excellent^a^ - Sufficient^b^ - Insufficient^c^ |
| *Skills:*  Be able to initiate strategies to reduce alcohol abuse. | Explain to the patient that there are different techniques to help reduce alcohol abuse. | Verbal information. | Oral communication. Visual graphics documents. | Skills of different techniques to reduce alcohol abuse.  Norm: 70-80% of participants.  Assessment tool:  Explain the different techniques. | - Excellent^a^ - Sufficient^b^ - Insufficient^c^ |
| *Attitudes:*  Start looking for help to reduce alcohol abuse. | Inform the patient where it can go to seek help and encourage them. | Counseling, nurse-patient communication and written support. | Oral communication, supported with the patient education handout. | Assess whether the patient has initiated strategies to achieve the objective described.  Norm: 60% of participants.  Assessment tool:  Interview 30-days (by telephone or at a follow-up visit) and 3 months (by telephone) after discharge. | Starts strategies?:   - - Yes   - No   Alcohol abuse?   - - Yes   - Consumption reduce   - No |

^a^ Reminds all information.

^b^ Reminds general information.

^c^ Not remember the information.
