# Supplementary material for: Impact of an Educational Program to Reduce Healthcare Resources in Community-Acquired Pneumonia: The EDUCAP Randomized Controlled Trial
Source: PLoS One. 2015 Oct 13;10(10):e0140202. doi: 10.1371/journal.pone.0140202 (PMC4603897; doi:10.1371/journal.pone.0140202)
Supplement: S3 File — (DOCX) [file pone.0140202.s003.docx]

**STUDY PROTOCOL**

**Impact of an Educational Program to Reduce Healthcare Resources in Community-Acquired Pneumonia: Study Protocol for a Multicenter, Randomized, Controlled Clinical Trial (EDUCAP).**

Authors: Jordi Adamuz,^1,2,3*^ Diego Viasus,^1,4^ Antonella Simonetti,^1^ Emilio Jiménez-Martínez,^1,2^ Lorena Molero,^1,2^ Maribel González-Samartino,^2,4^ Elena Castillo,^1,2^ María-Eulalia Juvé-Udina,^5,6^ María-Jesús Alcocer,^7^ Carme Hernández,^7^ María-Pilar Buera,^7^ Asun Roel,^7^ Emilia Abad,^7^ Adelaida Zabalegui,^7^ Pilar Ricart,^8^ Anna Gonzalez,^8^ Pilar Isla,^5^ Jordi Dorca,^9^ Carolina Garcia-Vidal,^1^ Jordi Carratalà^1,10^

*Correspondence: jordi.adamuz@gmail.com

^1^Department of Infectious Diseases, Hospital Universitari de Bellvitge, IDIBELL, Barcelona, Spain

^2^Department of Nursing, Hospital Universitari de Bellvitge, IDIBELL, Barcelona, Spain

^3^School of Health Science, Blanquerna-Ramon Llull University, Barcelona, Spain

^4^Clinical Research and Biotechnology Groups, Faculty of Medicine, Fundación Universidad del Norte, Hospital Universidad del Norte, Barranquilla, Colombia

^5^School of Nursing, University of Barcelona, IDIBELL, Barcelona, Spain

^6^Institut Català de la Salut, Barcelona, Catalunya, Spain

^7^Department of Nursing, Hospital Clínic de Barcelona, IDIBAPS, Barcelona, Spain

^8^Department of Nursing, Hospital Universitari Arnau de Vilanova, IRBLleida, Lleida, Spain

^9^Department of Respiratory Medicine, Hospital Universitari de Bellvitge, IDIBELL, Barcelona, Spain

^10^Faculty of Medicine, Department of Clinical Sciences, University of Barcelona,

Barcelona, Spain

**ABSTRACT**

**Introduction:** Recent studies have found that additional healthcare visits and rehospitalization after discharge are frequent among patients with community-acquired pneumonia (CAP). Researchers have recommended incorporating patient education to increase their understanding of post-discharge care. Notably, there is a lack of randomized, controlled trials examining the effects of an educational program in patients with CAP. The primary objective of this trial was to test whether the implementation of an individualized educational program for hospitalized patients with CAP would decrease the need for additional healthcare visits and readmissions within 30 days of hospital discharge. As secondary objectives, we evaluated the time to return to activities of daily living, the satisfaction with the information received and if the objectives were achieved.

**Methods and analysis:** The EDUCAP randomized, controlled, multicenter clinical trial was conducted between January 2011 and October 2014 in three Spanish hospitals. Hospitalized adults with CAP were included, but inmunocompromised patients, those derived from long-term care facilities, and those with cognitive deficits were excluded. Patients (n = 207) were randomly assigned to either an individualized educational program to improve their understanding of the disease and self-care management at home (n = 102) or a conventional information (n = 105). Demographic and clinical variables were collected by monitoring patients daily during hospitalization. Details of additional healthcare visits and rehospitalizations within 30 days of hospital discharge were obtained by searching the Healthcare Database of the Catalan Health Service. We evaluated patient satisfaction with the information provided during hospitalization after 30 days of hospital discharge. Furthermore, we assessed the educational program objectives were achieved before discharge and by 30 days and 90 days after hospital discharge using validated scales and questionnaires.

**Ethics and dissemination:** This trial will contribute to the development and implementation of educational programs during hospital admission among patients with CAP. Ethics approval was received from the Ethics Committee of Hospital Universitari de Bellvitge, Hospital Clínic de Barcelona, and Hospital Universitari Arnau de Vilanova.

**Trial Registration:** ControlledTrials.com identifier: ISRCTN39531840

**Keywords:** randomized trial, health education, community-acquired pneumonia, additional healthcare visits, and rehospitalization.

**INTRODUCTION**

In industrialized countries, community-acquired pneumonia (CAP) is a major cause of death, and it is the most frequent cause infectious mortality [1]. In addition, CAP accounts for more than 1 million hospitalizations annually, with costs exceeding $9.7 billion in United States [2] and 30-60% of patients diagnosed with CAP being admitted to hospital [3]. The high cost of treating CAP has raised interest in the development of strategies to reduce the length of hospitalization and increase the number of patients who receive care at home [4-6].

Recent studies have found that additional healthcare visits and rehospitalization within 30 days of discharge are frequent among patients with CAP [7,8]. Furthermore, it has been documented that hospitalization for this infection is associated with high long-term mortality compared with other major medical conditions [9-13]. Therefore, previous studies have concluded that new interventions are needed to lower the mortality rates and need for additional healthcare visits after hospital discharge [14,15].

Importantly, one qualitative study [16] found that most patients with pneumonia left hospital without a clear understanding of their disease, its treatment, or the follow-up required when they returned home. This led to an increase in primary care and emergency department visits, cost-ineffective inpatient bed allocation, and lower levels of patient satisfaction [16-19]. Indeed, previous studies identified that 7%-34% of patients with CAP required additional healthcare visits and rehospitalizations within 30 days of discharge [7,8,20,21]. This was mainly because of worsening clinical signs or symptoms of CAP and/or comorbidities [7]. The typical risk factors were patient comorbidities (mainly cardiopulmonary disease), lack of proper information before discharge and lifestyle factors (mainly unemployment, low education level, smoking, and alcohol abuse) [7,8,22]. In this regard, it has been suggested that some healthcare interactions might be preventable by adequately educated during discharge.

Discharge planning has been associated with improved referral to and utilization of post-discharge services, and therefore fewer readmissions possibly by preparing patients and caregivers for post-discharge care [23,24]. Although few studies have examined the effect of education-based interventions among CAP patients [16,19], they have concluded that discharge planning effectively improves patient knowledge, and results in cost-effective inpatient bed use, and increased patient satisfaction [16-19]. Significantly, not randomized controlled trials have examined the effects of an individualized educational program on additional healthcare visits after hospital discharge and rehospitalization in patients hospitalized with CAP.

We designed a randomized controlled trial to test the hypothesis that individualized educational program for hospitalized patients with CAP would decrease healthcare interactions after hospital discharge. The primary trial outcome was the composite frequency of additional healthcare visits and rehospitalization within 30 days of hospital discharge. Secondary outcomes included time to return to activities of daily living, the degree of patient satisfaction with information received, and the achievement of the program’s (i.e., patient fluid intake, adherence to drug therapy and preventive vaccines, knowledge and management of the disease, progressive adaptive physical activity, alcohol cessation, and smoking cessation).

**METHODS AND ANALYSIS**

The study was performed in three Spanish university hospitals between January 2011 and October 2014. The participating hospitals were: the infectious disease or respiratory disease departments of Hospital Universitari de Bellvitge, the respiratory disease department or home-care service of Hospital Clínic de Barcelona and the respiratory disease or internal medicine departments of Hospital Universitari Arnau de Vilanova de Lleida. The coordinating centre was Hospital Universitari de Bellvitge, which was responsible for handling clinical trial administrative authorization and regulatory affairs, professional training of participants, logistics, independent data, and safety monitoring, as well as general coordination and daily operational management of the trial for all of the participating sites.

**Selection and Enrolment**

All immunocompetent patients aged 18 years or older, diagnosed with CAP, and admitted to three participating hospitals were screened for eligibility. We excluded the following: patients from nursing homes or long-term care facilities; patients with neutropenia, immunoglobulin deficiencies, or HIV infection; who had undergone transplantation or splenectomy; and any patient receiving immunosuppressant and/or corticosteroid therapy (>20 mg/day of prednisone or equivalent). Furthermore, patients with cognitive deficit or those who did not understand Spanish or Catalan were excluded. Signed informed consent was obtained from all participants.

**Randomization**

A randomization assignment system allocated patients sequentially to receive either conventional information or an individualized educational program prior to hospital discharge. The aim of the individualized educational program was to improve the patient’s understanding of the disease and their self-care management at home, and was executed in two sessions before hospital discharge. Randomization was performed in computer-generated blocks of 10, with the randomization code kept by the clinical epidemiologist in a sealed envelope. The randomization was stratified by hospital. In the emergency department, patients who met the study criteria and provided written informed consent were randomized by a research nurse who opened the sealed, sequentially numbered, opaque envelopes. Blinding could not be carried out in this trial.

**Trial Intervention and Control**

The individualized educational program was performed according to the Precede model for assessing patient health needs and developing discharge planning [25]. One research nurse performed the individualized educational program in the intervention group. The patient, family, and/or caregiver received the program over two sessions of approximately 30 minutes each. In the first session, nurse assessed the patient’s health needs and provided an individualized educational program. Patient knowledge, skills, and attitudes about the program objectives were evaluated after last session. In addition, patients in the intervention group received an information leaflet about the self-management of CAP. These sessions were developed for the individual needs of each patient, to improve patient fluid intake, adherence to drug therapy and preventive vaccines, knowledge and management of the disease, and progressive adaptive physical activity, and also provided counseling for alcohol and smoking cessation if required. The interventions in the individualized educational program are fully detailed in a supplementary material.

The control group received conventional health promotion advice before hospital discharge that was not standardized between hospitals. Therefore, nurses and physicians provided advice based on their standard clinical practice. However, the conventional health promotion included information about special care to be followed at home, drug treatment, counseling about smoking and/or alcohol cessation, and information about healthcare visits. Nurses and/or physicians provided developed conventional information through the hospital discharge report. However, clinical staff did not know which patients were assigned to the conventional information group.

**Follow-up Protocol**

Patients were seen daily by their attending physicians and by at least one investigator during their hospital stay. The investigators assessed and recorded all primary and secondary outcome measures. Data collection was conducted: during hospitalization, after hospital at 30 days (during the discharge visit or by telephone) and at 90 days by telephone. Clinical and demographic variables were collected by daily monitoring of the patient during hospitalization. Information on additional healthcare visits and rehospitalizations within 30 days of hospital discharge was obtained by searching the admission databases of the three hospitals (Healthcare Database of the Catalan Health Service) and checked by asking patients at the final 30-day outpatient visit or by telephone. Patient satisfaction with the information received at discharge was evaluated 30-days after discharge. We evaluated whether or not the objectives had been achieved during hospitalization, and within 30 and 90 days of discharge by validated scales (Barthel scale and Haynes Sackett test) and specifically developed questionnaires to assess patient knowledge, skills, and attitudes. All assessments were made using a standard protocol with a checklist of items.

**Study Variables**

All baseline demographic variables, clinical variables, and primary/secondary outcomes (see, Table 1) were measured during hospitalization and within 30 and 90 days of hospital discharge.

**Outcome Measures**

The primary endpoint of the study was the composite frequency of additional healthcare visits and rehospitalizations within 30 days of discharge. This variable included the following: a) visits to a primary care centre because of doubts or complications related to CAP (scheduled follow-up visits were excluded); b) Emergency department visits for any reason; and c) Hospital readmission for any reason. These data were obtained by searching for hospital readmissions as described under the follow-up protocol. The province of Barcelona and Lleida provides universal health coverage for 5.8 million people [26]. All beneficiaries seen at hospitals in the Catalan Health Service are registered in the Systems, Applications & Products (SAP) Healthcare Database with a unique lifetime personal health number. Data on all hospitalizations and visits to primary care centre or emergency departments are routinely collected.

The secondary endpoint, time to return to normal activities or activities of daily living, was obtained by telephone enquiry of time off work and completion of the Barthel scale at 90 days of hospital discharge. Patient satisfaction with information received at discharge was evaluated after 30 days using the question “Are you satisfied with healthcare information regarding CAP received at discharge?” as previously reported. Responses were recorded on a Likert scale ranging from 1 to 5 (“very unsatisfactory” to “very satisfactory”). Patients were considered satisfied if the response recorded was 4 or 5.

To evaluate the efficacy of the individualized education program, we determined if the specific objectives of the educational program had been achieved prior to hospital discharge, 30 days after discharge (by visit or by telephone), and 90 days after discharge (by telephone) using validated scales and questionnaires. Patient fluid intake was evaluated by questioning. Adherence to drug therapy and preventive vaccines was evaluated by the Haynes Sacket test and the vaccination record. Knowledge and management of the disease was evaluated through a CAP knowledge test. Progressive adaptive physical activity was collected by the average daily time walking. Finally, we collected data on alcohol and smoking cessation or reduction during the three months after hospital discharge.

**Sample Size Calculation**

Sample size was calculated using the results of a Spanish cohort study about additional healthcare visits and rehospitalizations within 30 days of discharge in patients admitted with CAP in 2007-2009 in Hospital Universitari de Bellvitge. In this study, the rate of additional healthcare visits and rehospitalizations was 34.1% [7]. Using the chi-square bilateral test, we estimated a total sample size of 204 patients was needed to detect a 50% difference in additional healthcare visits and rehospitalizations between the two treatment groups, with an 80% power and a 5% significance level.

**Statistical Analysis**

Every effort was made to promote consistency between participating centers through independent monitoring visits to oversee trial progress and ensure that it was conducted, recorded, and reported in accordance with the protocol and relevant CONSORT guidelines [27].

Clinical trial data will be summarized using descriptive statistics and demographic and clinical data were analyzed. A descriptive analysis of continuous variables was performed (i.e., n, mean, standard deviation, range, and median). The 95% frequency confidence intervals were also presented if appropriate.

A bivariate analysis was performed to compare primary and secondary outcomes in the two groups overall as well as for each hospital. A bivariate analysis, using the chi-square test or the Fisher exact test, was used for categorical variables. For quantitative variables, the Mann Whitney *U* or Student *t* test were used depending on the results of the Kolmogorov Smirnov test for normality. In addition, percentage differences for each outcome and the mean differences between the two groups were calculated with corresponding 95% confidence intervals.

Data for the primary and secondary end points were analyzed on an intention-to-treat and per protocol basis. The intention-to-treat analysis included all randomly assigned patients. The statistical analysis was performed with version 18.0 of the SPSS software package (SPSS Inc., Chicago, Illinois). Statistical significance was established at an *α* value of 0.05. All reported *P* values were based on two-tailed tests.

**Safety and Adverse Event Reporting**

This trial only implemented an individualized educational program in patients admitted with CAP. Therefore, the potential for adverse events were very limited. In accordance with Good Clinical Practice (GCP), we carefully monitored all adverse events occurring during the study (until observed by the investigator or reported by the participant), whether or not they were attributed to the educational program. Causality was assessed by the principal investigator and re-evaluated by a qualified physician from the infectious diseases department of the Hospital Universitari de Bellvitge.

**Project Management**

Strategic management of the trial was the responsibility if the Scientific Coordination Team (SCT), comprising the trial coordinating investigator and the local research staff. The SCT was responsible for supervising trial enrolment and patient follow-up, offering practical clinical advice to assist local teams with and medical aspects of the study implementation. The SCT was also in charge of data management and statistical analysis.

The Institut d’Investigació Biomèdica de Bellvitge (IDIBELL) provided the facilities for developing the clinical trial at the coordinating trial site.

Operational management of the study was the responsibility of the Central Management Team (CMT), comprising the EDUCAP project coordinator, the clinical researcher, the infectious diseases physician, and the assisting research staff working at the Clinical Trial Unit. The CMT was responsible for handling clinical trial administrative authorization and regulatory affairs, day-to-day trial operational management, and independent data and safety monitoring.

**Data and Safety Monitoring**

The aim of monitoring was to ensure patient protection, data quality, and trial integrity. To ensure that investigators were following the protocol, complying with regulatory and GCP standards, and collecting and reporting quality data, a clinical researcher appointed by the Trial CMT was given responsibility for supervising study progress at each site. Monitoring involved periodic on-site visits and centralized supervision activities to identify, prevent, or mitigate risks to data quality, patient protection, and trial integrity.

**Ethic, Deontological and Regulatory Considerations**

Investigators ensured that the study was conducted in accordance with the principles of the Declaration of Helsinki and the International Conference for Harmonization (ICH) Guidelines for GCP. The study was performed in full conformity with the applicable regulations.

The protocol, informed consent form, participant information sheets, and any applicable documents received full ethical and regulatory approval in writing. The trial was registered in a publicly accessible database (the EU *Clinical Trials Register).* All substantial amendments to the original approved documents obtained further approval from the relevant Ethics Committee.

If patients met the inclusion criteria, investigators obtained written informed consent prior to study enrolment. In all cases, we provided appropriate information about the effects, objectives, methods, anticipated benefits, and potential risks of participation. The investigators also explained the right to withdraw consent at any time and for any reason. If a patient was unable to read or write, a legal representative was present during the informed consent process and signed the consent form on the patient’s behalf.

The trial staff ensured that participant confidentiality was preserved. Participants were only identified by an identification code on the case report form. All study documents and data were stored securely, and were only accessible to the principal investigator and authorized staff for trial related purpose. Data from case report forms were encrypted in an SPPS database in the custody of the Trial’s CMT.

The CONSORT [27] guidelines were followed when publishing the study results in clinical journals and presenting them at national and international conferences.

**DISCUSSION**

The need for additional healthcare visits and rehospitalization within 30 days of discharge is frequent among patients with CAP, and has been documented in 34% of patients [7,8]. A recent study found that the main reasons for such interactions were clinical deterioration in CAP and other chronic comorbid conditions [7]. Furthermore, it has been shown that hospitalization in this context is associated with high long-term mortality compared with other major medical conditions [9-13], with rates of 11% to 53%. The increase in long-term mortality appears to be related to several aspects, although chronic comorbid conditions are paramount [9,11,12]. Taking these factors into account, it has been concluded that new interventions are needed to lower mortality and additional healthcare interactions after hospital discharge in patients with CAP [14,15].

A qualitative study [16] has reported that most patients leave hospital without a clear understanding of pneumonia, its treatment, or the follow-up required when they return home, which leads to an increase in primary care and emergency department visits, cost-ineffective inpatient bed use, and lower levels of patient satisfaction [17,18]. Moreover, it is argued that strategies to reduce the length of hospitalization and to manage CAP in the community requires an increased emphasis on the information and support needs of patients at home [16-19,28].

To date, few researchers have examined the effect of educational interventions in CAP patients [16,19]. One study comparing interventions between consecutive cohorts of patients hospitalized for CAP. Using a series of interventions designed to improve patient knowledge and experience with care, they assessed the impact on the time to clinical stability as measured by the times to the switch from intravenous to oral antibiotics and to discharge. Importantly, they were able to reduce the time on intravenous antibiotics from 5 days to 4 days. Their patient education leaflet explained that it takes time to recover from pneumonia and recommended taking prescribed medications, eating healthy foods, and monitoring for warning signs such as fever. Physicians, nurses, and social workers received training on the application of the guidelines and educated patients about pneumonia and post-discharge care. Patients who responded to the survey reported that they received all the information needed for their recovery (pre-intervention, 75%; post-intervention, 94%) and more reported that they were able to spot the danger signals of relapse (46% vs 60%). The study concluded that educational interventions improved patient experiences and increased their understanding of post-discharge care. Therefore, they recommended incorporating patient education into efforts to streamline inpatient care [19].

Conversely, a pre-post test study evaluated a multifactorial intervention to improve the quality and efficiency of inpatient pneumonia care and patient understanding. They developed evidence-based treatment guidelines and critical pathways, conducted educational sessions with physicians, distributed pocket reminder cards, promoted standardized orders, and developed bilingual patient education materials. To improve the patient understanding of the illness, they used a patient education handout. The intervention led to an increase in the use of recommended antimicrobial therapy and decreases the proportion of patients being discharged prior to becoming clinically stable. However, there were no improvements in the time to first dose of antibiotics, time to discharge, or patient education outcomes. This study concluded that patient education had no effect on resource use or patient knowledge [29].

Studies to date are limited by their time series design without concurrent controls or randomization. In addition, they did not assess if the individualized objectives of the educational program were achieved, and they did not evaluate the effects of the intervention on healthcare interactions after hospital discharge. Therefore, the results lack generalizability to other settings. Nevertheless, the data do provide encouragement for further research to provide solid evidence for the implementation of an educational intervention in patients with CAP to improve patient management after discharge and to decrease healthcare visits and rehospitalizations within 30 days of discharge.

In conclusion, given the evidence on this topic, the adequate design of the present protocol, and the efforts invested to meet the regulatory requirements for clinical trials were expected to result in reliable and valid evidence about the effects of educational interventions in patients with CAP. Furthermore, the results of this study should contribute to the development and implementation of in-hospital educational programs for patients with CAP, where the goals are to decrease healthcare visits and rehospitalizations within 30 days of discharge and improve self-management.

**Trial Status**

The EDUCAP trial recruitment period was closed in July 2014, with 207 participants. Long-term follow-up visits were completed in October 2014.

**List of abbreviations**

CAP: Community-acquired pneumonia; CMT: Central Management Team; GPC: Good Clinical Practice; ICH: International Conference Harmonization; IDIBELL: Institut d’Investigació Biomèdica de Bellvitge; SCT: Scientific Coordination Team.

**Competing Interests**

The authors declare that they have no competing interests.

**Funding and Sponsorship**

The trial is supported by the Ministerio de Ciencia e Innovación, Instituto de Salud Carlos III, co-financed by the European Development Regional Fund ‘A Way to Achieve Europe’, the Spanish Network for Research in Infectious Diseases (REIPI RD06/0008), the Fondo de Investigación Sanitaria de la Seguridad Social (grant 07/0864), and the Institut d’Investigació Biomèdica de Bellvitge.

**Acknowledgements**

We are grateful to all involved in the trial, the clinical teams, the investigators, the patients, the families, the caregivers, and the coordination teams of Hospital Universitari de Bellvitge, Hospital Clínic de Barcelona and Hospital Universitari Arnau de Vilanova de Lleida.

**EDUCAP Study Group: Participating Hospitals and Investigators:**

*Hospital Universitari de Bellvitge***:** Jordi Adamuz (PI), Diego Viasus, Antonella Simonetti, Emilio Jiménez-Martínez, Lorena Molero, Maribel González-Samartino, Elena Castillo, Maria-Eulàlia Juvé-Udina, Pilar Isla, Jordi Dorca, Carolina Garcia-Vidal, Jordi Carratalà.

*Hospital Clínic de Barcelona:* María-Jesús Alcocer (PI), Carme Hernández, María-Pilar Buera, Asun Roel, Emi Abad, Adela Zabalegui.

*Hospital Universitari Arnau de Vilanova de Lleida:* Pilar Ricart (PI), Anna Gonzalez.

**EDUCAP Scientific Coordination Team:**

Jordi Adamuz^*^, Emilio Jiménez-Martínez^*^, Lorena Molero^*^, Elena Castillo^*^.

**EDUCAP General Management Team:**

Diego Viasus^*^, Jordi Carratalà^*^, Carolina García-Vidal^*^, Pilar Isla^*^.

^*^Hospital Universitari de Bellvitge.

**Author Contributions**

JA, DV, PI, and CGV were responsible for formulating the overall research question and the study design. JA was the coordination investigator and Scientific Coordination Team Leader. DV was the General Management team coordinator and the qualified researcher of the infectious diseases department. JC and DV provided input to revised versions of the protocol and other study documents, and supervised the first draft of this manuscript. CGV, and MEJU provided input to revised versions of the protocol and other study documents, and supervised the trial sample logistics and analysis. PI and AZ provided expertise regarding the development of the individualized education program. JA, AS, EJM, LM, MGS, EC, MJA, CH, MPB, EA, AR, PR, AG, and JD participated in patient recruitment and monitoring. All authors provided input into the drafting of the manuscript, and read and approved the final version.**References:**

1. Mandell LA, Wunderink RG, Anzueto A, et al. Infectious Diseases Society of America/American Thoracic Society consensus guidelines on the management of community-acquired pneumonia in adults. *Clin Infect Dis* 2007; 44 Suppl 2: S27–72.
2. Lave J, Lin C. The cost of treating patients with community-acquired pneumonia. *Semin Respir Crit Care Med* 1999; 20(3):189–97.
3. Carratalà J. Outpatient care or hospitalization? A crucial decision in the treatment of community-acquired pneumonia. *Enferm Infecc Microbiol Clin* 2004; 22(2):61–3.
4. Carratalà J, Fernández-Sabé N, Ortega L, et al. Outpatient care compared with hospitalization for community-acquired pneumonia: a randomized trial in low-risk patients. *Ann Intern Med* 2005; 142(3):165–72.
5. Garcia-Vidal C, Carratalà J, Díaz V, et al. Factors associated with prolonged hospital stay in community-acquired pneumonia. *Enferm Infecc Microbiol Clin* 2009; 27(3):160–4.
6. Halm EA, Fine MJ, Kapoor WN, et al. Instability on hospital discharge and the risk of adverse outcomes in patients with pneumonia. *Arch Intern Med* 2002; 162(11):1278–84.
7. Adamuz J, Viasus D, Campreciós-Rodríguez P, et al. A prospective cohort study of healthcare visits and rehospitalizations after discharge of patients with community-acquired pneumonia. *Respirology* 2011; 16(7):1119–26.
8. Jasti H, Mortensen EM, Obrosky DS, Kapoor WN, Fine MJ. Causes and risk factors for rehospitalization of patients hospitalized with community-acquired pneumonia. *Clin Infect Dis* 2008; 46(4):550–6.
9. Mortensen EM, Metersky ML. Long-term mortality after pneumonia. *Semin Respir Crit Care Med* 2012; 33(3):319–24.
10. Mortensen EM, Kapoor WN, Chang C-CH, Fine MJ. Assessment of mortality after long-term follow-up of patients with community-acquired pneumonia. *Clin Infect Dis* 2003; 37(12):1617–24.
11. Johnstone J, Eurich DT, Majumdar SR, Jin Y, Marrie TJ. Long-term morbidity and mortality after hospitalization with community-acquired pneumonia: a population-based cohort study. *Medicine (Baltimore)* 2008; 87(6):329–34.
12. Yende S, Angus DC, Ali IS, et al. Influence of comorbid conditions on long-term mortality after pneumonia in older people. *J Am Geriatr Soc* 2007; 55(4):518–25.
13. Bruns AHW, Oosterheert JJ, Cucciolillo MC, et al. Cause-specific long-term mortality rates in patients recovered from community-acquired pneumonia as compared with the general Dutch population. *Clin Microbiol Infect* 2011; 17(5):763–8.
14. Ewig S, Torres A. Community-acquired pneumonia as an emergency: time for an aggressive intervention to lower mortality. *Eur Respir J* 2011; 38(2):253–60.
15. Restrepo MI, Faverio P, Anzueto A. Long-term prognosis in community-acquired pneumonia. *Curr Opin Infect Dis* 2013; 26(2):151–8.
16. Baldie DJ, Entwistle VA, Davey PG. The information and support needs of patients discharged after a short hospital stay for treatment of low-risk Community Acquired Pneumonia: implications for treatment without admission. *BMC Pulm Med* 2008; 8:11.
17. Hansen HE, Bull MJ, Gross CR. Interdisciplinary collaboration and discharge planning communication for elders. *J Nurs Adm* 1998; 28(9):37–46.
18. Rose KE, Haugen MB. Discharge planning: your last chance to make a good impression. *Medsurg Nurs* 2010; 19(1):47–50, 53.
19. Horowitz CR, Chassin MR. Improving the quality of pneumonia care that patients experience. *Am J Med* 2002; 113(5):379–83.
20. Capelastegui A, España Yandiola PP, Quintana JM, et al. Predictors of short-term rehospitalization following discharge of patients hospitalized with community-acquired pneumonia. *Chest* 2009; 136(4):1079–85.
21. Dagan E, Novack V, Porath A. Adverse outcomes in patients with community acquired pneumonia discharged with clinical instability from Internal Medicine Department. *Scand J Infect Dis* 2006; 38(10):860–6.
22. Torres A, Peetermans WE, Viegi G, Blasi F. Risk factors for community-acquired pneumonia in adults in Europe: a literature review. *Thorax* 2013; 68(11):1057–65.
23. Haddock KS. Collaborative discharge planning: nursing and social services. *Clin Nurse Spec* 1994; 8(5):248–52, 288.
24. Houghton A, Bowling A, Clarke KD, Hopkins AP, Jones I. Does a dedicated discharge coordinator improve the quality of hospital discharge? *Qual Health Care* 1996; 5(2):89–96.
25. Mazloomymahmoodabad S, Masoudy G, Fallahzadeh H, Jalili Z. Education based on precede-proceed on quality of life in elderly. *Glob J Health Sci* 2014; 6(6):36649.
26. Instituto Nacional de Estadística website: Censo de Población [Internet] 2014 [Accessed Aug 9, 2014]. Available from: http://www.ine.es/jaxi/tabla.do
27. Schulz KF, Altman DG, Moher D. CONSORT 2010 statement: updated guidelines for reporting parallel group randomised trials. *Int J Surg* 2011; 9(8):672–7.
28. Johnson A, Sandford J. Written and verbal information versus verbal information only for patients being discharged from acute hospital settings to home: systematic review. *Health Educ Res* 2005; 20(4):423–9.
29. Halm EA, Horowitz C, Silver A, Fein A, Dlugacz YD, Hirsch B, et al. Limited impact of a multicenter intervention to improve the quality and efficiency of pneumonia care. *Chest* 2004; 126(1):100–7.

TABLES

| **Table 1**. EDUCAP study variables | |
| --- | --- |
| **Demographic data** | Sex, age, education levels, nationality, support at home, current/former smoker or drinker, Influenza vaccination (<1 year), Pneumococcal vaccination (<5 years) and previous CAP (<1 year) |
| **Clinical data** | Complications during hospitalization, etiology, ICU admission, clinical stability on hospital discharge^a^, length of hospital stay (days), CURB-65, Charlson comorbidity index, 30-day mortality |
| **Main outcomes** | Additional healthcare visits and rehospitalizations within 30 days of discharge:   - Visits to a primary care centre because of doubts or complications related to CAP (scheduled follow-up visits were excluded) - Emergency department visits for any reason - Hospital readmission for any reason |
| **Secondary outcomes** | Time to return to normal activities or activities of daily living within 90 days of hospital discharge:   - Time off work - The Barthel scale |
|  | Satisfaction degree with information received at discharge (30-days of hospital discharge) |
|  | Objectives of educational program (30 and 90 days of hospital discharge):   - Patient fluid intake ≥ 2 liters daily - Adherence to drug therapy and preventive vaccines: Haynes Sackett test and vaccination schedule - Knowledge and management of the disease: CAP knowledge test^b^ - Progressive adaptive physical activity: average daily time walking ≥20 minutes - Alcohol and smoking cessation |
| Abbreviations: CAP, community-acquired pneumonia; ICU, intensive care unit; CURB-65 (confusion, urea >7 mmol/L, respiratory rate ≥ 30/min, low systolic [<90mm hg] or diastolic [<60 mm hg] blood pressure, age ≥ 65 years).  ^a^ Clinical stability was defined as Halm et al.  ^b^ Test developed by Adamuz et al. for the study. | |
